# 免疫组化法检测*EML4-ALK*融合突变价值的*meta*分析

**DOI:** 10.3779/j.issn.1009-3419.2016.01.05

**Published:** 2016-01-20

**Authors:** 畅 刘, 璐 蔡, 殿胜 钟, 竞 王

**Affiliations:** 1 300052 天津，天津医科大学总医院肿瘤科 Department of Medical Oncology, Tianjin Medical University General Hospital, Tianjin 300052, China; 2 300052 天津，天津医科大学总医院，天津市肺癌研究所 Tianjin Lung Cancer Institute, Tianjin Medical University General Hospital, Tianjin 300052, China

**Keywords:** 免疫组化, 肺肿瘤, *EML4-ALK*, 融合基因, *Meta*分析, Immunohistochemistry, Lung neoplasms, *EML4-ALK*, Fusion gene, *Meta* analysis

## Abstract

**背景与目的:**

棘皮动物微管相关蛋白4(echinoderm microtubule-associated protein 4, *EML4*)与间变性淋巴瘤激酶(anaplastic lymphatic tumor kinase, *ALK*)重排形成的融合基因存在于大约5%的非小细胞肺癌(non-small cell lung cancer, NSCLC)患者中，是继表皮生长因子受体(epidermal growth factor receptor, *EGFR*)、*K-ras*之后又一新型靶点基因。有数据显示携带*EML4-ALK*融合基因的NSCLC患者接受ALK抑制剂治疗后，其疾病控制的有效率可达80%，探索和建立能够准确快速检测出NSCLC患者*EML4-ALK*融合突变的方法，是筛选出适合治疗的优势人群的关键。本研究分析免疫组化法(immunohistochemistry, IHC)检测*EML4-ALK*融合基因突变的敏感度与特异度，评价该方法准确性及临床应用价值，从而为肺癌患者"个体化分子治疗"提供依据。

**方法:**

通过Pubmed数据库检索所有符合检索条件的文献，末次检索日期为2015年2月25日，根据纳入和排除标准进行进一步筛选，采用诊断试验*meta*分析方法，比较特异性抗体免疫组化法与"金标准"荧光原位杂交(fluorescence *in situ* hybridization, FISH)法的敏感度、特异度，以明确特异性抗体IHC作为筛查方法的可行性。

**结果:**

本文11篇文献纳入*meta*分析，*EML4-ALK*融合基因免疫组化累计病例3, 234例，诊断比值比(diagnositic odds ratio, DOR)为1, 135.00(95%CI: 337.10-3, 821.46)；综合受试者工作特征曲线(summary receiver operating characteristic curve, SROC)下面积为0.992, 3(SEAUC=0.003, 2)，Q^*^统计量为0.964, 4 (SEQ^*^=0.008, 7)。

**结论:**

特异性抗体IHC法检测*EML4-ALK*融合基因的方法可行，具有高特异度和敏感度，可作为一种简单快速的筛查方法，具有临床应用价值。

## Abstract (en)

**Background and objective:**

Te fusion between echinoderm microtubule-associated protein 4 (*EML4*) and anaplastic lymphatic tumor kinase (*ALK*) rearrangement is present in approximately 5% of non-small cell lung cancer (NSCLC) patients. It has been regarded as another new target gene afer epidermal growth factor receptor (*EGFR*) and K-ras. Figures showed that the disease control rate could reach up to 80% in NSCLC patients with *EML4-ALK* fusion gene afer treated with ALK inhibitors. Tus, exploring an accurate and rapid detecting method is the key in screening NSCLC patients with *EML4-ALK* expressions. Te aim of this study is to analyze the specifcity and sensitivity of IHC in detecting *EML4-ALK* fusion mutations. To evaluate the accuracy and clinical value of this method, and then provide basis for individual molecular therapy of NSCLC patients.

**Methods:**

Using Pubmed database to search all documents required. Te deadline of retrieval was February 25, 2015. Ten further screening the articles according to the inclusion and exclusion criteria. Using diagnostic test *meta*-analysis methods to analyze the sensitivity and specifcity of the immunohistochemistry (IHC) method compared with fluorescence *in situ* hybridization (FISH) method.

**Results:**

Eleven literatures were added into the *meta* analysis, there were 3, 234 of total cases. Te diagnositic odds ratio (DOR) was 1, 135.00 (95%CI: 337.10-3, 821.46); the area under curve (AUC) of summary receiver operating characteristic curve (SROC) curve was 0.992, 3 (SEAUC=0.003, 2), the Q^*^ was 0.964, 4 (SEQ^*^=0.008, 7).

**Conclusion:**

Immunohistochemical detection of *EML4-ALK* fusion gene mutation with specifc antibody is feasible. It has high sensitivity and specifcity. IHC can be a simple and rapid way in screening *EML4-ALK* fusion gene mutation and exhibits important clinical values.

表皮生长因子受体(epidermal growth factor receptor, EGFR)和K-ras作为最早被研究的治疗靶点，已经涌现出许多针对这些靶点的制剂，并已进入临床，取得较好的临床效果^[1-3]^。随着研究的不断深入，继上述靶点之后，非小细胞肺癌(non-small cell lung cancer, NSCLC)中又出现一新型靶点基因，即棘皮动物微管相关蛋白4-间变性淋巴瘤激酶(echinoderm microtubule-associated protein 4-anaplastic lymphatic tumor kinase, *EML4-ALK*)融合基因。*EML4-ALK*是在NSCLC患者肿瘤标本中由Soda等^[4]^于2007年首次发现，进一步探索表明，该基因是由2号染色体短臂内转位inv(2)(p21p23)的EML4与ALK融合而成^[5-7]^。

针对ALK的抑制剂克唑替尼已通过美国食品药品监督管理局(Food and Drug Administration, FDA)批准应用于肺癌患者的Ⅲ期临床研究中^[8]^，Shaw等^[9]^在第47届美国临床肿瘤学会(American Society of Clinical Oncology, ASCO)大会上对其研究的报告表示克唑替尼可明显提高患者的生存率。探索和建立能够准确快速检测出NSCLC患者*EML4-ALK*融合突变的方法，将有助于筛选出适合ALK-TKI治疗的优势人群，为肺癌患者的个体化靶向治疗提供依据。

免疫组化法(immunohistochemistry, IHC)操作简便，是实验室最普遍适用的蛋白质的筛查与诊断方法^[10]^。该方法可以在不损伤细胞内部结构特征的前提条件下，通过检测肿瘤细胞内特异性抗原的表达，从而与正常组织相鉴别，以确定有无*ALK*融合基因的表达^[11, 12]^。另外IHC法还可以同时将正常组织与病理组织的形态结构特征进行鉴别，实现快速筛选的过程^[13]^。已有研究^[14, 15]^发现应用IHC法对*EML4-ALK*融合基因突变进行检测的结果，经荧光原位杂交(fluorescence *in situ* hybridization, FISH)和逆转录聚合酶链反应(reverse transcriptase-polymerase chain reaction, RT-PCR)法验证后，其结果具有高度的一致性。本文通过诊断试验*meta*分析的方法，比较特异性抗体免疫组化法与"金标准"FISH法的敏感度、特异度，以明确特异性抗体IHC作为筛查方法的可行性及临床应用价值。

## 1 材料与方法

### 1.1 筛选文献资料

按照以下检索词通过英文数据库pubmed检索所有符合的文献。检索的关键词为：EML4-ALK、immunohistochemistry、NSCLC、FISH。中文检索通过中国期刊全文数据库，检索关键词：肺癌、EML4-ALK、免疫组化。末次检索日期为2015年2月25日。

### 1.2 文献资料纳入标准

使用兔单克隆抗体D5F3(VENTANA)检测*EML4-ALK*融合基因存在情况，同时应用检测金标准FISH法比较其敏感度和特异度的文献。①研究对象采用FISH法为金标准，被检测者所患肺癌的病理类型需要在文献中注明；②可以在文章中根据已提供的数据计算出各个研究案例的真阳性、假阳性、真阴性、假阴性或者文章中已明确计算出上述各个数值；③参加研究的各个组中的案例数均应超过20；④研究类型为含有兔单克隆抗体D5F3(VENTANA)检测对NSCLC患者*EML4-ALK*融合基因检测价值的回顾性或前瞻性研究；⑤文献中*EML4-ALK*融合基因的检测通过可以进行评价的具有统一标准的免疫组化方法及标准(IHC阳性定义：5%以上肿瘤细胞的细胞质着色定义为阳性，FISH法标本由NSCLC患者的经福尔马林固定和石蜡包埋标本获得)。

### 1.3 文献排除标准

具有以下之一标准者排除该项研究。①文献中未规定统一判定标准的免疫组化法检测*EML4-ALK*融合基因；②反复模型实验中，文献中的样本量偏小或者发表时间较早也应排除；③IHC与除此之外的另一些检验方法(如RT-PCR法)比较，而无FISH对照的文献，主要因为RT-PCR法虽为临床上较为常见的检测手段，虽敏感度较高，但仍无法检测出未知的突变，并且对于RNA的质量有较高的要求，临床上的组织标本大多数是经过福尔马林的固定、蜡块包埋处理后，RNA易大面积解体，导致其灵敏度的减低，此外很难避免的是由于扩增所致的假阳性结果，这些对于免疫组化法的阴性预测值(negative predictive value, NPV)、阳性预测值(positive predictive value, PPV)的结果统计均会造成一定的干扰；④综述、社论、摘要、评论。

### 1.4 异质性分析

根据受试者工作特征曲线(receiver operating characteristic curve, ROC)平面上由各个研究的精准估测量所绘制出的图像是否与"肩臂"状的典型分布相一致，从而对由阈值效应所导致的异质性对各个研究对象逐一分析；通过*Q*检验对由非阈值效应所导致的异质性在各个研究对象逐一鉴定，之后依据异质性的分析结果来筛选出适宜的统计学试验模型，以完成下一步的*meta*分析。

### 1.5 *Meta*分析

*Meta*分析对比特异性抗体IHC法与FISH比较的特异度、敏感度，对此方法的准确性进行评价判定。整理并综合各个试验方法的最初数据(真阴性、真阳性、假阴性、假阳性的例数)，使用DerSimonian Laird随机效应模型计算兔单克隆抗体D5F3(VENTANA)免疫组织化学方法的特异度和敏感度的平均值，以及95%置信区间(confidence interval, CI)和各自比值。将SROC曲线通过Mose's constant线性模型进行拟合，通过Q统计量、诊断比值比(diagnositic odds ratio, DOR)以及曲线下面积(aera under curve, AUC)对NSCLC患者IHC法检测*EML4-ALK*融合基因存在情况的准确度进行评价。将曲线的X轴定义为此次*meta*分析中所录入的各个研究对象的(1-特异度)，Y轴定义为敏感度，从而勾画出SROC曲线，通过直接视觉感官对诊断实验的准确性进行评估，曲线下所围成的面积越大，曲线越接近于左上角，则具有较高的诊断准确性。本文统计用软件为Meta-Disc 1.4，以*P* < 0.05为差异有统计学意义。

## 2 结果

### 2.1 检索结果及纳入文献

依照所列出的检索关键词进行初级检索，共找到59篇文献。排除摘要和阅读文题16篇，初步纳入所需文献43篇。深入阅读全文，将不符合录入标准的文献排除共25篇，排除具有重复内容的文献共2篇，排除所需初始数据不能完全获取的文献共5篇，最终共有11篇文献符合纳入标准，如图 1。

**1:**
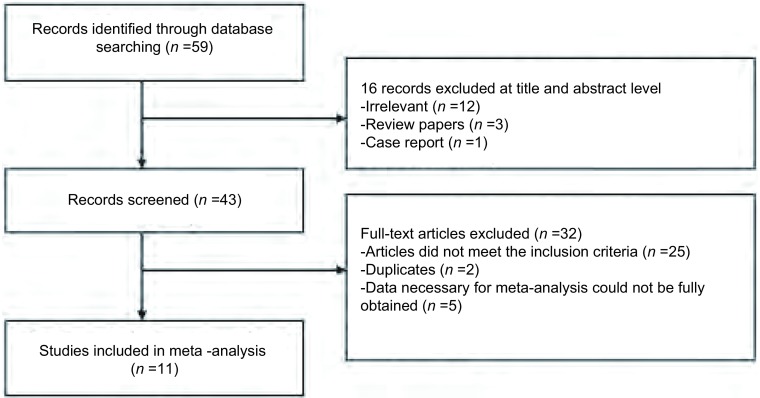
文献选择流程图 Flow chart for study selection

### 2.2 纳入研究的基本特征和质量评价

本论文共纳入11项研究，*EML4-ALK*融合基因免疫组化累计病例3, 234例，各研究IHC法特异度、敏感度及例数参见表 1。

**1:** 纳入文献的样本数及免疫组化方法的敏感度及特异度 Samples of included studies and the sensitivity and specificity by IHC

	*n*	IHC	FISH	Sensitivity (%)	Specificity (%)
Alì G 2014^[16]^	523	18	20	74.0	99.0
Sullivan HC 2015^[17]^	110	11	7	100.0	96.0
Hutarew G 2014^[10]^	303	128	14	100.0	60.6
Guo L 2014^[18]^	404	29	29	100.0	100.0
Zhou JY 2013^[19]^	253	26	20	100.0	97.4
Demidova I 2014^[20]^	36	1	1	100.0	100.0
Zhu X 2014^[21]^	525	27	10	100.0	96.7
To KF 2013^[22]^	373	22	20	100.0	99.4
Zhang YG 2013^[23]^	473	20	15	100.0	98.9
Han XH 2013^[24]^	139	45	43	100.0	97.9
Zhang MX 2014^[25]^	95	8	4	100.0	95.6
IHC: immunohistochemistry; FISH: fluorescence *in situ* hybridization.					

### 2.3 异质性检验

效应量将DOR作为参照标准，分析特异性抗体D5F3免疫组化与荧光标记法FISH的异质性，Q检验显示Cochran-Q为16.5，*P* > 0.05，*I*^2^为39.4%，ALK抗体免疫组化研究间不存在异质性，本文分析选用随机效应模型。

### 2.4 *Meta*分析

应用特异性抗体IHC法的特异性、敏感度和似然比。图 2所示为EML4-ALK特异性抗体对NSCLC诊断敏感度的森林图。ALK抗体鉴别NSCLC患者*EML4-ALK*融合基因的平均敏感度为0.99(95%CI: 0.96-1.00)。图 3所示为EML4-ALK特异性抗体对NSCLC诊断特异度的森林图。ALK抗体鉴别*EML4-ALK*融合基因的平均特异度为0.95(95%CI: 0.94-0.96)。图 4所示为阳性似然比(positive likelihood ratio, PLR)为50.64(95%CI: 8.77-292.61)。图 5所示为EML4-ALK特异性抗体对NSCLC诊断*EML4-ALK*融合基因的阴性似然比(negative likelihood ratio, NLR)为0.06(95%CI: 0.03-0.12)。图 6所示为EML4-ALK特异性抗体对NSCLC诊断*EML4-ALK*融合基因的DOR为1, 135.00(95%CI: 337.10-3, 821.46)。图 7所示为EML4-ALK特异性抗体对NSCLC诊断*EML4-ALK*融合基因的SROC曲线。其AUC为0.992, 3(SEAUC=0.003, 2)，Q^*^统计量为0.964, 4 (SEQ^*^=0.008, 7)。

**2:**
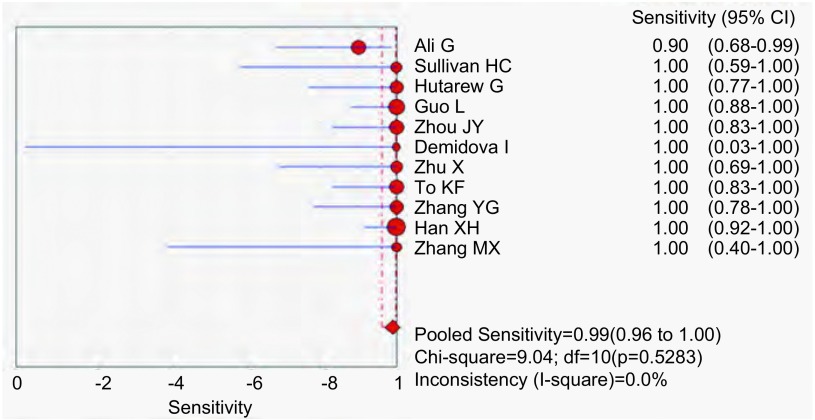
EML4-ALK抗体敏感度森林图 The forest plots of EML4-ALK antibody sensitivity; EML4 -ALK : echinoderm microtubule-associated protein 4-anaplastic lymphatic tumor kinase.

**3:**
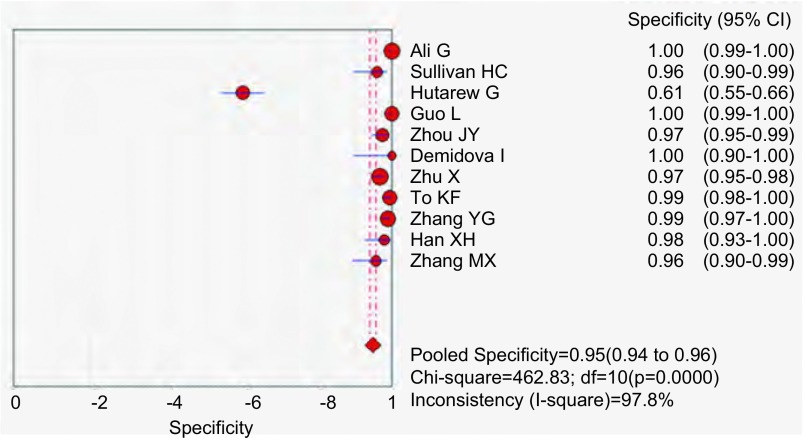
EML4-ALK抗体特异度森林图 The forest plot of EML4-ALK antibody specificity

**4:**
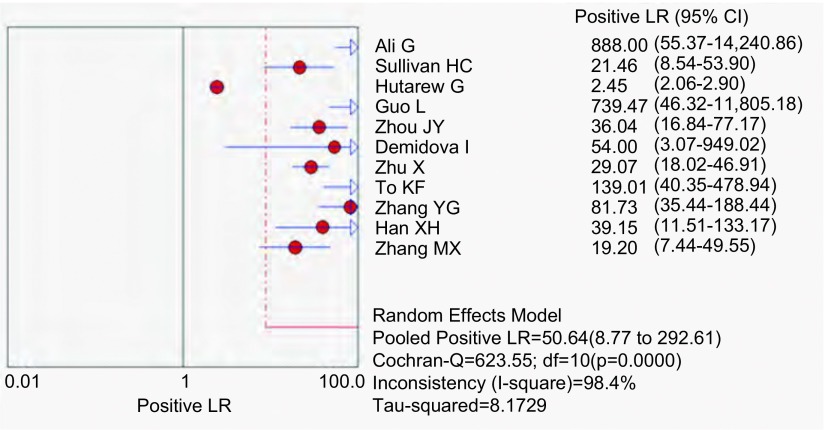
EML4-ALK抗体PLR森林图 The forest plot of EML4 -ALK antibody positive likelihood ratio (PLR)

**5:**
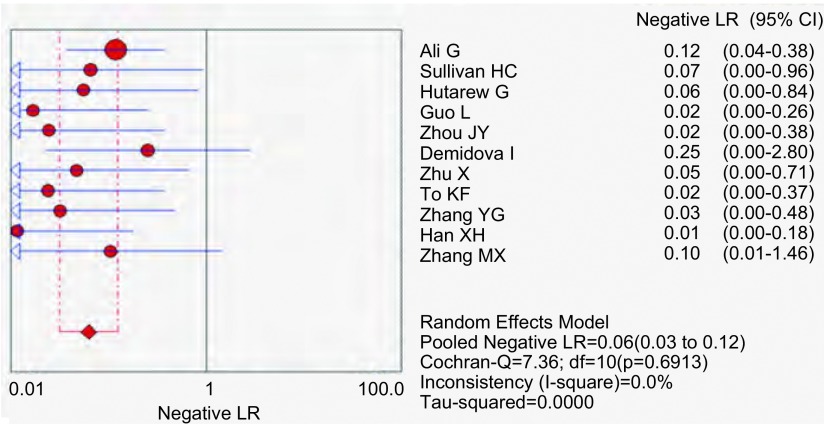
EML4-ALK抗体NLR森林图 The forest plot of EML4-ALK antibody negative likelihood ratio (NLR)

**6:**
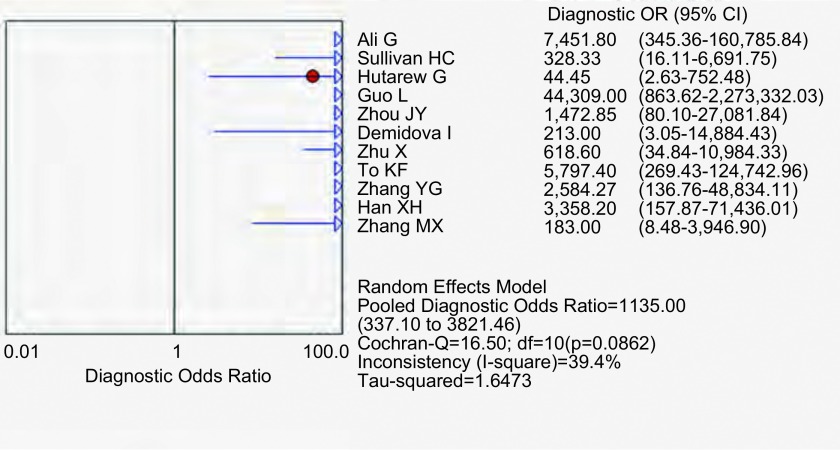
EML4-ALK抗体DOR森林图 The forest plot of EML4-ALK antibody diagnositic odds ratio (DOR)

**7:**
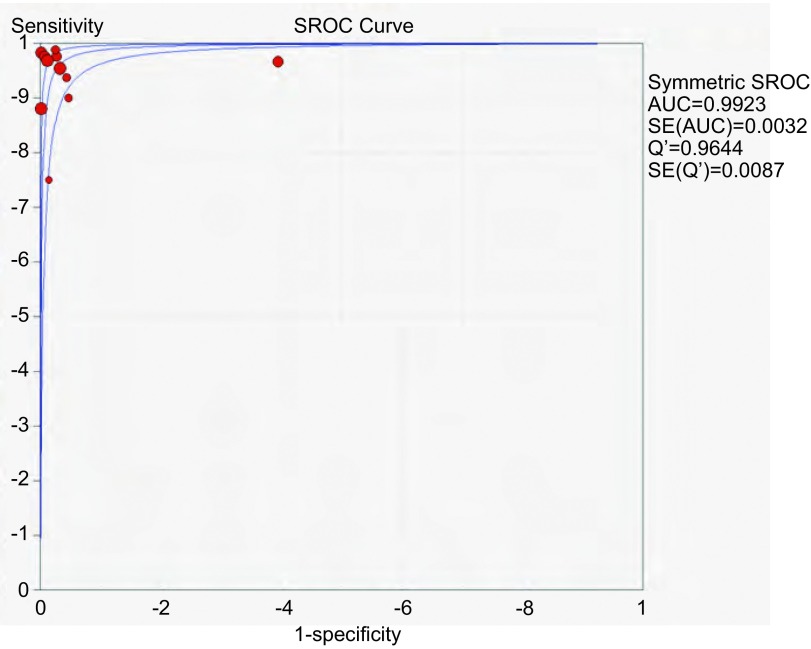
EML4-ALK抗体的SROC曲线 The summary receiver operating characteristic curve (SROC) curve of EML4-ALK antibody

### 2.5 异质性分析

由EML4-ALK特异性抗体的SROC曲线可见，各研究对应的点散在分布，呈"肩臂"状外观，分别计算(1-特异性)对数与灵敏度对数的*Spearman*相关系数ρ，EML4-ALK特异性抗体的ρ值为-0.126，*P* > 0.05，显示无阈值效应存在。

综上所述，EML4-ALK特异性抗体免疫组化法检测*EML4-ALK*融合基因，该方法灵敏度高，特异度较高，可靠易行，免疫组化法在对融合基因的筛检方面具有高度的可行性，可作为临床上一种有应用价值的检测手段。

## 3 讨论

本文通过*meta*分析对录入的11项研究进行整合，根据SROC曲线的拟合及诊断效应量的合并，将ALK特异性抗体免疫组化法与免疫荧光原位杂交法对*EML4-ALK*融合基因的诊断效能进行比较。结果显示ALK特异性抗体鉴别NSCLC患者EML4-ALK的平均特异度为0.95(95%CI: 0.94-0.96)，平均敏感度为0.99(95%CI: 0.96-1.00)。综合分析结果提示免疫组化法具有较高的敏感性与特异性，参考相关文献^[26, 27]^报道，不同研究的特异性和敏感性多波动在80%-100%和90%-100%。本文结论与相关文献相符。

通常情况下可以认为，当满足NLR < 0.1或PLR > 10时，大体上能够明确或者除外某项诊断^[28]^。本研究得出的ALK特异性抗体诊断*EML4-ALK*融合基因的NLR为0.06(95%CI: 0.03-0.12)，PLR为50.64(95%CI: 8.77-292.61)，这一结果显示免疫组化结果不论呈现为阴性或阳性，均可作为一种辅助手段，指导临床医师对某项疾病做出判断，具有广泛的临床应用前景。

DOR是对诊断试验的分析结果和某种疾病关联度的一种反映指标。当其值在1以下时，提示正常人可能比阳性患者通过诊断性试验判定为阳性的概率更大；当其值等于1时，提示正常人和阳性患者无法经诊断性试验被区分；当其值在1以上时，表示此诊断性试验的鉴别效果随数值的增大而更好^[29]^。本研究中ALK特异性抗体诊断*EML4-ALK*融合基因的DOR为1, 135.00(95%CI: 337.10-3, 821.46)，表明诊断性试验的鉴别诊断结果良好。

本研究将符合录入标准的11篇文献所记录的完整数据，计算整合NLR、PLR、DOR、特异度和敏感度，描绘SROC曲线，SROC曲线又称作综合受试者工作特征曲线，无异质性的干扰，能够整合特异度和灵敏度的分析结果^[30]^。从整体上对诊断试验的准确度进行评定，该曲线的X坐标轴为1-特异度，Y坐标轴为灵敏度，理论依据为使FRO与TRP之间的非线性关系经过FRP、TRP的Logit变换后转换成为一种线性关系，采用最小乘法对各项参数进行统计分析，得出评估诊断性试验准确程度的统计结果，同时列出SROC曲线回归方程。分析本研究的SROC曲线表明，ALK特异性抗体的AUC为0.992, 3，Q^*^统计量为0.964, 4，曲线接近左上角，曲线下所围成的面积范围大，表明上述特异性抗体免疫组化法鉴别*EML4-ALK*融合突变的准确度较高。本研究所录入的个体对象间不存在异质性，表明研究间的变异仅由于抽样误差造成，无需进一步做*meta*回归，即探索异质性来源的可能因素。

本文*meta*分析的不足：①录入的研究对象具有局限性：通过盲法测查以及盲法评判特异性抗体免疫组化法的诊断性试验，从而尽可能降低诊断的倾向性，然而大部分文献报道未标明检测方法是否应用了盲法，有可能会出现检测结果发生偏倚；②*Meta*分析具有局限性：筛选获得的文献尚不全面。数据库的检索范围仅限于已公开发表的文献报道，一些未发表的文章无法获取，如会议论文，对于一些灰色文献存在漏检的可能性；检索文献的语言类型仅限于英文和中文，除此之外的其他语言类型可能会被漏检；③纳入本*meta*分析的样本量较少，需要更进一步的大样本量来验证结论。

综上所述，特异性抗体免疫组化法检测*EML4-ALK*融合基因突变的诊断准确度高，操作简便，具有一定的临床应用前景，且随着特异性抗体的发展，IHC将有可能成为NSCLC患者*EML4-ALK*融合基因突变检测的常规筛查程序，可首选作为一种简单标准的NSCLC患者快速检测方法。
